# Mycobacterium Ulcerans Treatment – Can Antibiotic Duration Be Reduced in Selected Patients?

**DOI:** 10.1371/journal.pntd.0003503

**Published:** 2015-02-06

**Authors:** Raquel Cowan, Eugene Athan, N. Deborah Friedman, Andrew J. Hughes, Anthony McDonald, Peter Callan, Janet Fyfe, Daniel P. O’Brien

**Affiliations:** 1 Department of Infectious Diseases, Barwon Health, Geelong, Australia; 2 Department of Plastic Surgery, Barwon Health, Geelong, Australia; 3 Victorian Infectious Diseases Reference Laboratory, Melbourne, Australia; 4 WHO Collaborating Centre for *Mycobacterium ulcerans*, VIDRL, Melbourne, Australia; 5 Manson Unit, Mèdecins Sans Frontières, London, United Kingdom; 6 Department of Medicine and Infectious Diseases, Royal Melbourne Hospital, University of Melbourne, Melbourne, Australia; Foundation Raoul Follereau, FRANCE

## Abstract

**Introduction:**

*Mycobacterium ulcerans* (*M. ulcerans*) is a necrotizing skin infection endemic to the Bellarine Peninsula, Australia. Current treatment recommendations include 8 weeks of combination antibiotics, with adjuvant surgery if necessary. However, antibiotic toxicity often results in early treatment cessation and local experience suggests that shorter antibiotic courses may be effective with concurrent surgery. We report the outcomes of patients in the Barwon Health *M. ulcerans* cohort who received shorter courses of antibiotic therapy than 8 weeks.

**Methodology / Principal findings:**

A retrospective analysis was performed of all *M. ulcerans* infections treated at Barwon Health from March 1, 1998 to July 31, 2013. Sixty-two patients, with a median age of 65 years, received < 56 days of antibiotics and 51 (82%) of these patients underwent concurrent surgical excision. Most received a two-drug regimen of rifampicin combined with either ciprofloxacin or clarithromycin for a median 29 days (IQR 21–41days). Cessation rates were 55% for adverse events and 36% based on clinician decision. The overall success rate was 95% (98% with concurrent surgery; 82% with antibiotics alone) with a 50% success rate for those who received < 14 days of antibiotics increasing to 94% if they received 14–27 days and 100% for 28–55 days (p<0.01). A 100% success rate was seen for concurrent surgery and 14–27 days of antibiotics versus 67% for concurrent surgery and < 14 days of antibiotics (p = 0.12). No previously identified risk factors for treatment failure with surgery alone were associated with reduced treatment success rates with < 56 days of antibiotics.

**Conclusion:**

In selected patients, antibiotic treatment durations for *M. ulcerans* shorter than the current WHO recommended 8 weeks duration may be associated with successful outcomes.

## Author Summary

Buruli ulcer is a necrotizing skin and subcutaneous tissue infection caused by *Mycobacterium ulcerans* and is the third most common mycobacterial infection, behind tuberculosis and leprosy, world-wide. In recent years, the World Health Organisation has modified its guidelines for *M*. *ulcerans* treatment, moving from predominantly surgical to predominantly medical based management. It now recommends the combination of oral rifampicin and intramuscular streptomycin for a period of eight weeks as first-line therapy, with surgery as adjunctive therapy if necessary. The Barwon Health experience from south-eastern Australia has demonstrated that the entirely oral combination of rifampicin with either ciprofloxacin or clarithromycin for eight weeks can be an effective treatment option. However, these antibiotics are often toxic leading to early cessation, especially in the elderly. In addition, clinicians have been using a shorter duration of therapy for smaller lesions that have also been surgically managed. This study reviews our experience treating *M*. *ulcerans* with antibiotic durations of less than 8 weeks and demonstrates that successful outcomes can be achieved in selected patients, with success rates influenced by the duration of treatment and the use of surgical excision. This finding needs confirmation in further studies, but could have significant benefits in terms of reducing toxicity and improving adherence associated with Buruli ulcer antibiotic treatment.

## Introduction

*Mycobacterium ulcerans* (*M*. *ulcerans)* infection, or Buruli ulcer, is a necrotizing infection of the skin and subcutaneous tissue and is the third most common mycobacterial infection in immunocompetant people after tuberculosis and leprosy. It has been reported in 33 predominantly subtropical and tropical countries, with west and central Africa being the worst affected regions [1]. In Australia endemic foci of infection are found in tropical Far North Queensland and temperate regions of southern Victoria [2].

Up until 2004, the World Health Organisation (WHO) recommended wide surgical excision as treatment for *M*. *ulcerans* lesions, with no role for antibiotics which were thought to be ineffective [3]. However surgical treatment was often difficult to access in resource-limited settings [4], was limited by high recurrence rates [5, 6] and in severe cases caused significant cosmetic morbidity and increased costs [7, 8]. Mounting clinical evidence of the effectiveness of antibiotics [9–14] has resulted in a paradigm shift to a predominantly medical approach. The latest WHO recommendations are for eight weeks combination antibiotic therapy with intramuscular streptomycin and oral rifampicin. Surgical intervention is recommended only to hasten healing of more extensive ulcers, if antibiotics are contraindicated or not tolerated, or at a patient’s request. In addition, if surgery is required, an initial four weeks of antibiotics prior to surgery is recommended [2, 15].

Clinicians at Barwon Health began using antibiotics to treat *M*. *ulcerans* infections from the Bellarine Peninsula in 1998 [5]. Since this time the proportion of patients receiving antibiotics has increased, resulting in fewer recurrences and permitting more conservative, and thus less reconstructive, surgery [12]. Oral antibiotics have subsequently reduced hospitalisations and the cost of treatment [7]. Our current treatment practice for the majority of *M*. *ulcerans* lesions is an oral combination of rifampicin with either ciprofloxacin or clarithromycin for eight weeks. Surgery is used for debriding necrotic wounds or closing large tissue defects to increase the rate of wound healing, for patients unable or unwilling to take antibiotics, and for those preferring the more rapid healing of small lesions that surgical excision and direct closure enables compared with the often prolonged healing of lesions treated with antibiotics alone [2].

However, these protracted antibiotic treatment regimens are not without toxicity, and 16–33% of patients, often elderly, cease them early due to side effects [12, 13]. In addition, shorter courses of adjunctive antibiotics (4–6 weeks) have been offered for smaller lesions treated with surgical excision and direct closure based on previous experience where cure was obtained for 100% of 21 patients who received between 12 and 30 days of antibiotics combined with surgical excision [12].

The aim of this study was to review the experience and outcomes of patients in the Barwon Health *M*. *ulcerans* observational cohort who received a shorter course of antibiotic therapy than the standard recommended duration of 8 weeks.

## Methods

A retrospective analysis was performed of all confirmed *M*. *ulcerans* infections treated at Barwon Health from March 1, 1998 to July 31, 2013. Data was collected prospectively using Epi-info 6 (CDC Atlanta, USA). Definitions used for the study can be found in Table 1.

**Table 1:** Parameter definitions used during analysis.

Item	Definition
*M*. *ulcerans* case	Lesion clinically consistent with *M*. *ulcerans PLUS*
	- Positive *M*. *ulcerans* PCR or culture* from a swab, biopsy or excised lesion
	- Histopathology of an excised lesion showing a necrotic ulcer with the presence of acid-fast bacilli consistent with acute *M*. *ulcerans* infection
Surgical definitions	- Major surgery: closure of an excised lesion using a split skin or full thickness skin graft or vascularised tissue flap
	- Positive margins: histology of excised tissue showing granulomatous inflammation or necrotic tissue extending to the tissue margins
Long-course antibiotics	Duration of antibiotics ≥ 56 days
Short-course antibiotics	Duration of antibiotics < 56 days
Treatment success	Healing of the entire lesion without recurrence 12 months after the antibiotic treatment course had been completed.
Treatment failure	A recurrent *M*. *ulcerans* lesion within 12 months of treatment completion that was either a) culture positive for *M*. *ulcerans* or b) had histopathology showing a necrotic ulcer with the presence of acid-fast bacilli consistent with acute *M*. *ulcerans* and did not show evidence of a paradoxical reaction

*Mycobacterial cultures were performed using Lowenstein–Jensen media and incubated for 12 weeks

Patients who had received antibiotics for *M*. *ulcerans* were included in the study. Treatment duration was separated into ≥ 56 days (long-course therapy), and < 56 days (short-course therapy). Patients were followed for 12 months after the cessation of treatment. Drug dosages for adults included rifampicin 10 mg/kg/day (up to a maximum of 600 mg daily), ciprofloxacin 500 mg twice daily, moxifloxacin 400 mg once daily, clarithromycin 500 mg twice daily and ethambutol 15 mg/kg/day. A complication of medical therapy was defined as an adverse event attributed to an antibiotic that required its cessation. Immune suppression was defined as current treatment with immunosuppressive medication (eg. prednisolone) or active malignancy. The position of a *M*. *ulcerans* lesion was described as distal if it was on or below the elbow or knee.

Surgical specimens were sent to the Victorian Infectious Diseases Reference Laboratory (VIDRL) for mycobacterial culture using Lowenstein-Jensen media and a 12-week incubation period.

Data was analysed using STATA 13 (StataCorp, Texas, USA). The main variable assessed was treatment outcome and defined as success or failure as outlined in Table 1. Proportions were compared using 2x2 tables and the Chi-squared and Fisher’s exact test. Median values were compared using the Mann-Whitney test.

### Ethics

This is an observational, retrospective cohort study with analysis performed on anonymised data. Verbal patient consent, or consent from a parent or guardian in the case of a minor, was gained for collection of data and noted in the patient medical record. Barwon Health’s Human Research and Ethic Committee have approved this analysis.

## Results

### Demographics

From March 1, 1998 to July 31, 2013 there were 252 patients diagnosed with *M*. *ulcerans* infection at Barwon Health. Forty-one patients who were not treated with antibiotics were excluded from this analysis, two were excluded due to unrelated deaths during follow-up and two were lost to follow-up. Therefore 207 patients were included; 110 (53%) were male and the median age was 59 years (IQR 36–75 years). Sixty-two (30%) patients received less than 56 days of antibiotic treatment (short-course therapy) whilst 145 (70%) patients had 56 days or more (long-course therapy) (Table 2).

**Table 2:** Baseline characteristics of patients receiving short-course (< 56 day) or long-course (≥ 56 days) antibiotics for *M*. *ulcerans* infection.

	Short-course (n = 62)	Long-course (n = 145)	p value
***Sex***			
Male	25 (40%)	85 (59%)	p = 0.02
Female	37 (60%)	60 (41%)	
***Age***			
< 60 years	24 (39%)	80 (55%)	p = 0.03
≥ 60 years	38 (61%)	65 (45%)	
***Immune suppression***			
No	54 (87%)	137 (94%)	p = 0.07
Yes	8 (13%)	8 (6%)	
***Diabetes***			
No	54 (87%)	133 (92%)	p = 0.30
Yes	8 (13%)	12 (8%)	
***Type of lesion***			
Ulcerative	56 (90%)	121 (83%)	p = 0.07
Nodular	5 (8%)	6 (4%)	
Plaque	0 (0%)	2 (1%)	
Oedematous	1 (2%)	16 (11%)	
***WHO category***			
1	57 (92%)	113 (77%)	p = 0.003
2	0 (0%)	24 (17%)	
3	5^@^ (8%)	9 (6%)	
**Lesion Position**			
Proximal	10 (16%)	13 (9%)	p = 0.13
Distal	52 (84%)	133 (91%)	
Duration of symptoms prior to diagnosis*			
≤ 75 days	46 (78%)	110 (77%)	p = 0.92
> 75 days	13 (22%)	32 (23%)	
***Positive margins^#^***			
No	23 (48%)	15 (19%)	p<0.001
Yes	25 (52%)	65 (81%)	
***Major surgery^&^***			
No	25 (49%)	25 (30%)	p = 0.03
Yes	26 (51%)	57 (70%)	

^@^ 1 patient with an oedematous lesion, 3 patients with 2 lesions and 1 patient with 13 lesions

*n = 200 (missing data in 7 patients)

^#^n = 128

^&^n = 133

### Comparisons of patient characteristics for short (n = 62) and long-course (n = 145) antibiotic groups

Those receiving short-course antibiotics were more likely to be female, ≥ 60 years of age, have WHO category 1 lesions, and were less likely to have had major surgery or have positive margins (Table 2). However there were no significant differences in the proportions that were diabetic, the type or site of lesions, or the duration of symptoms prior to diagnosis (Table 2).

### Short-course treatment cohort (n = 62)

**Fig 1:**
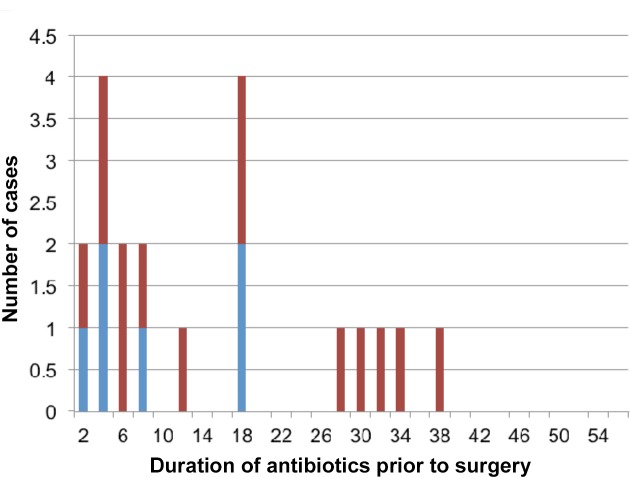
Red column-Negative tissue culture. Blue column-Positive tissue culture.

The median age of the patients was 65 years (range 2–94 years) and their lesion was present for a median of 42 days prior to diagnosis (IQR, 24–70 days). Fifty-one (82%) patients had their lesions surgically excised, with the defect directly closed in 25 (49%) cases, and closed with split skin grafts in 21 (41%), a vascularised tissue flap in 4 (8%) and both a split skin graft and a vascularised tissue flap in 1 case (2%). Thirty-nine (76%) of those who had surgery had previously commenced antibiotics for a median of 8 days (IQR, 4–18 days). Twenty of these patients had specimens sent for *M*. *ulcerans* culture and their median duration of antibiotics prior to surgery was 12 days (IQR 5–28 days). Six of these patients (30%) had positive mycobacterial cultures; 4 had received 8 days or less of antibiotics, one had received 17 days and one 18 days of antibiotics prior to surgery (Fig. 1).

The regimens used were predominantly rifampicin-based in combination with ciprofloxacin in 41 (66%), clarithromycin in 12 (19%), clarithromycin/ethambutol in 3 (5%) and moxifloxacin in 2 (3%) patients. Other combinations included clarithromycin with either ethambutol in 2 patients (3%) or ciprofloxacin (1 patient; 2%) and clarithromycin alone in one patient (2%). The median duration of treatment was 29 days (IQR 21–41 days). Cessation secondary to side effects, often attributed to more than one of the drugs in a combination, occurred in 34 (55%) patients. The most common side effects included nausea, vomiting, diarrhoea, hepatitis, rash, joint aches and acute renal failure. In twenty-two (36%) patients the clinician’s decision to cease treatment was based on presumed adequate treatment when combined with surgical management of the lesions. Two patients ceased due to interactions with concomitant medications or patient’s wishes in each of the surgically treated and antibiotic only groups.

### Overall treatment results (n = 62)

**Fig 2:**
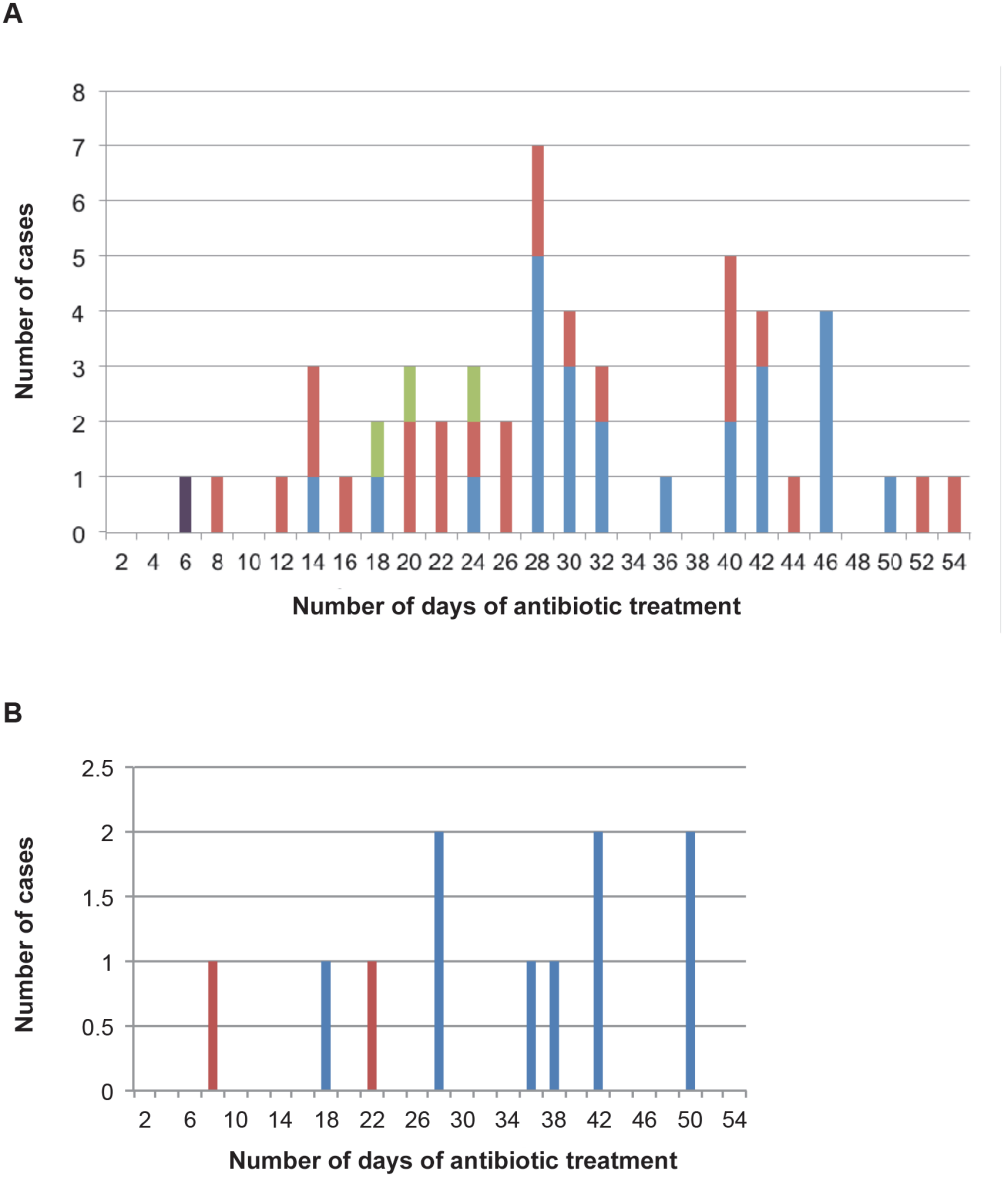
A) Purple column-Positive margin / treatment failure. Green column-Unknown margin / treatment success. Red column -Negative margin / treatment success. Blue column-Positive margin / treatment success. B) Red column-Treatment failure. Blue column-Treatment success.

Fifty-nine of 62 (95%) short-course patients experienced treatment success, including the 5 WHO category 3 patients. Details of the three patients who failed treatment are listed in Table 3. This compares with a 99% success rate (144/145) for patients who received long-course treatment. Outcomes according to the duration of antibiotic treatment are presented in Table 4 and Fig. 2. Overall, those who received less than 14 days of therapy had a 50% success rate which increased to 94% if they received 14–27 days of treatment and 100% for 28–55 days treatment (p<0.01; Table 4, Fig. 2A). The success rate for those receiving < 28 days compared to ≥ 28 days was reduced (86% versus 100%, p = 0.04).

**Table 3:** Characteristics of patients who failed short-course antibiotic treatment for *M*. *ulcerans* infection.

Year	Age/Sex	Co-morbid	Lesion	Sx	Abx	Days	Cessation reason	Result
2001	79F	Diabetes	Distal leg ulcer	No	ClarithromycinEthambutol	7	Nausea	Local recurrence
2002	73F	Diabetes I/S^	Distal wrist nodule	No	Rifampicin Clarithromycin Ethambutol	21	Hepatitis	Local recurrence
2006	94F	Nil noted	Distal leg ulcer	Yes*	Rifampicin Ciprofloxacin	6	Nausea / diarrhoea	Local and distal (ankle) recurrence

Sx = surgery; Abx = antibiotics

^Immunosuppressed on prednisolone for eczema

*positive surgical margin

**Table 4:** Proportion of patients achieving treatment success according to antibiotic treatment duration for *M*. *ulcerans* infection.

	Days of antibiotic treatment				p value
	1–13	14–27	28–41	42–56	
***Overall (n = 62)***					
Treatment success	2 (50%)	17 (94%)	28 (100%)	12 (100%)	0.001
Treatment failure	2 (50%)	1 (6%)	0 (0%)	0 (0%)	
***Surgery + antibiotics (n = 51)***					
Treatment success	2 (67%)	16 (100%)	23 (100%)	9 (100%)	0.001
Treatment failure	1 (33%)	0 (0%)	0 (0%)	0 (0%)	
***Antibiotics alone (n = 11)***					
Treatment success	0 (0%)	1 (50%)	5 (100%)	3 (100%)	0.05
Treatment failure	1 (100%)	1 (50%)	0 (0%)	0 (0%)	

### Surgery plus antibiotics (n = 51)

There was a 98% (50/51 cases) treatment success rate in those receiving antibiotics and surgery (Table 4, Fig. 2). Cure rates were significantly increased in those who received longer durations of therapy (p<0.001). All 23 patients treated with antibiotics and surgery for 14–28 days achieved treatment success, in comparison to 67% (2 patients) if they received antibiotics for less than 14 days (p = 0.12). When only cases with positive margins were included the cure rates remained higher for treatment durations of 14–28 days compared with < 14 days (8/8 patients versus 0/1 patients, p = 0.11).

### Antibiotics alone (n = 11)

Although the numbers are small, there was an 82% (9/11 cases) success rate for those treated with antibiotics alone. Cure rates were significantly increased in those who received a duration of therapy greater than 28 days (p = 0.05). Two of three patients receiving antibiotics alone for less than 28 days failed treatment, whilst the 6 patients receiving 28–42 days of treatment were cured (p = 0.08; Table 4; Fig. 2B).

### Success rates with respect to known risk factors for recurrence with surgery alone

There were no significant differences in recurrence rates for those patients with positive margins, age ≥ 60 years, duration of symptoms > 75 days, immunosuppression or distal lesions (Table 5).

**Table 5:** Proportion of patients achieving treatment success for *M*. *ulcerans* infection in high-risk categories.

Parameter	No per group	No treatment success (%)	p value
Positive surgical margins	25	24 (96)	0.33
Negative surgical margins	23	23 (100)	
Age < 60 years	24	24 (100)	0.28
Age ≥ 60 years	38	35 (92)	
Duration of symptoms ≤ 75 days	46	44 (96)	0.53
Duration of symptoms > 75 days	13	12 (92)	
Immunosuppressed	6	5 (83)	0.27
Not immunosuppressed	56	54 (96)	
Distal lesion	52	49 (94)	1.0
Proximal lesion	10	10 (100)	

## Discussion

Our study suggests that an antibiotic treatment course for *M*. *ulcerans* infections of less than the current WHO recommended of 8 weeks duration can be associated, in selected patients, with good outcomes with a success rate of 95% overall and 98% in patients who received combined medical and surgical therapy. However the effectiveness of short-course therapy was influenced by the duration of antibiotics. Success rates of 100% were achieved after at least 28 days of antibiotics but this reduced to 94% if 14–27 days of antibiotics were used and only 50% if less than 14 days of antibiotics were used (p = 0.001).

Surgery may have also influenced success rates with improved results in those who received antibiotics plus surgery compared to antibiotics alone. Although the numbers were small in the group treated with antibiotics alone (11 patients), it may be that shorter antibiotic treatment courses are effective when there has been a reduction of bacterial load by surgery. This finding needs to be further investigated in studies involving larger patient numbers.

These outcomes with increased patient numbers build on our previous findings from this cohort of 100% treatment success in 21 patients receiving 12–30 days of antibiotics combined with surgical excision [12]. The overall success rate of 98% in this study is similar to that reported from our Barwon Health cohort treated with antibiotics alone for at least 56 days (36 patients; 97% success rate)[13] or at least 60 days of antibiotics plus surgery (44 patients; 100% success rate) [12], and in the long-course group reported in this study (99%). This suggests that in our observational cohorts similar success rates are possible for short-course treatment compared to when antibiotics have been used for at least 56 days.

These success rates are also comparable to studies from African cohorts using 56 days of antibiotics. In a randomised trial in Ghana, 8 weeks of rifampicin and streptomycin or 4 weeks of rifampicin and streptomycin followed by 4 weeks of oral rifampicin and clarithromycin reported no recurrences after 12 months in 147 patients [9]. In Benin an 8-week combination of clarithromycin and rifampicin in 30 patients, with half undergoing surgery, resulted in no treatment failures [10]. In a separate study in Ghana, 8 weeks of rifampicin and streptomycin resulted in no recurrences at one year in 158 patients, of whom only 8 had adjunctive surgical treatment [11]. Finally, in an observational trial in Ghana involving 43 patients with lesions less than 15cm, using 2 weeks of rifampicin and streptomycin followed by 6 weeks of rifampicin and clarithromycin, 93% had completely healed their lesions and there were no recurrences out to one year [14].

Supportive evidence for the effectiveness of short-course treatment is provided by the inability in our study to culture *M*. *ulcerans* from surgical specimens taken after more than 18 days of antibiotics (Fig. 1). Further Australian experience reported by Gordon and colleagues [16] showed that excisional samples from 2 patients were culture negative after 4 or 6 weeks of rifampicin combined with moxifloxacin or clarithromycin. Similar findings have also been reported from a WHO-sponsored study of 21 African patients treated with oral rifampicin and intramuscular streptomycin where *M*. *ulcerans* could not be cultured from 11 excised lesions after 4 weeks of antibiotic treatment [17]. Furthermore, all samples except one were culture negative after 4 weeks of rifampicin and clarithromycin in 9 patients in Benin [10].

Of note however, in a recent study by Phillips and colleagues [14], where patients were treated with 2 weeks of rifampicin and streptomycin followed by 6 weeks or oral rifampicin and clarithromycin, 3 out of 7 lesions were culture positive 12 weeks after initiation of treatment and 4 weeks after cessation of therapy, albeit at a lower bacterial loads. However despite this all the lesions healed and there were no recurrences at 12 months. Furthermore, Nienhuis and colleagues showed that 3 of 5 lesions healed without surgical debridement after being culture positive post treatment with 4 weeks of standard therapy followed by 4 weeks of rifampicin and clarithromycin [9]. This suggests that sterilization of tissue cultures during antibiotic treatment may not be required to achieve successful treatment outcomes and this further supports the potential for antibiotic treatment durations to be shortened. Spontaneous healing of lesions without treatment has also been reported [18] and it may be possible that once antibiotics +/- surgery have reduced the bacterial load the immune system can sterilize any persisting infection.

In our study, the majority of patients in the short-course group were selected because of antibiotic side-effects requiring a cessation in treatment. If severe side-effects relate to increased serum antibiotic levels compared to those not experiencing such reactions, it is possible that this may have contributed to enhanced microbiological efficacy and improved rates of cure with a reduced antibiotic course.

A limitation of this study is its observational design where patients were not randomised into short and long-course treatment groups. Therefore there were differences in baseline characteristics between the groups that may have influenced outcomes. Firstly there was a higher proportion of patients with WHO category 1 lesions in the short-course group. If smaller lesions with a lower bacterial load require less antibiotic treatment this may have positively influenced outcomes in this group. Secondly the short-course group had a lower proportion of patients with positive margins which may favour improved outcomes as they are associated with a higher rate of failure in the absence of antibiotic treatment [19]. Conversely the short-course group had a higher proportion of patients with immunosuppression and age > 60 years, which have been associated with an increased rate of surgical treatment failure in the absence of antibiotics and may have negatively affected outcomes in this group [19]. Of note in our study, none of the factors associated with treatment failure for surgery alone were associated with treatment failure with the use of short-course compared to long-course antibiotic therapy (Table 5). Furthermore the majority of patients received fluoroquinolone antibiotics in combination with rifampicin and results therefore may not be generalisable to patients not receiving fluoroquinolone-containing regimens. Finally, the lack of randomization also means that there may have been other unmeasured confounders that could have influenced outcomes.

Our study represents a selected subset group of patients from south-eastern Australia. The results may not be generalisable to all *M*. *ulcerans* lesions and other settings such as Africa where a higher proportion of patients present late with advanced disease (WHO category 2 and 3) [11], cohorts are younger, the proportion of ulcerative lesions is less, and there is an incidence of HIV co-infection [20]. Nevertheless, based on our findings, we advocate for further research into the effectiveness of shorter antibiotic regimens for the treatment of *M*. *ulcerans*, including prospective randomised trials comparing them against standard 8-week treatment regimens. Shorter regimens could potentially include 4 weeks of antibiotics for surgically-excised lesions and 6 weeks of antibiotics if they are used alone. If successful, this could have significant benefits in terms of reducing toxicity and improving adherence associated with *M*. *ulcerans* antibiotic treatment.

### Conclusions

In selected patients, antibiotic treatment durations for *M*. *ulcerans* shorter than the current WHO recommended 8 weeks duration may be associated with successful outcomes. Success may be influenced by the duration of treatment and the use of surgical excision.

## Supporting Information

S1 Checklist STROBE Checklist. (DOC)
